# iSeq: an integrated tool to fetch public sequencing data

**DOI:** 10.1093/bioinformatics/btae641

**Published:** 2024-10-24

**Authors:** Haoyu Chao, Zhuojin Li, Dijun Chen, Ming Chen

**Affiliations:** Department of Bioinformatics, College of Life Sciences, Zhejiang University, Hangzhou 310058, China; Key Laboratory of Pharmaceutical Biotechnology, School of Life Sciences, Nanjing University, Nanjing 210023, China; Key Laboratory of Pharmaceutical Biotechnology, School of Life Sciences, Nanjing University, Nanjing 210023, China; Department of Bioinformatics, College of Life Sciences, Zhejiang University, Hangzhou 310058, China

## Abstract

**Motivation:**

High-throughput sequencing technologies [next-generation sequencing (NGS)] are increasingly used to address diverse biological questions. Despite the rich information in NGS data, particularly with the growing datasets from repositories like the Genome Sequence Archive (GSA) at NGDC, programmatic access to public sequencing data and metadata remains limited.

**Results:**

We developed iSeq to enable quick and straightforward retrieval of metadata and NGS data from multiple databases via the command-line interface. iSeq supports simultaneous retrieval from GSA, SRA, ENA, and DDBJ databases. It handles over 25 different accession formats, supports Aspera downloads, parallel downloads, multi-threaded processes, FASTQ file merging, and integrity verification, simplifying data acquisition and enhancing the capacity for reanalyzing NGS data.

**Availability and implementation:**

iSeq is freely available on Bioconda (https://anaconda.org/bioconda/iseq) and GitHub (https://github.com/BioOmics/iSeq).

## 1 Introduction

Next-generation sequencing (NGS) has revolutionized molecular biology by enabling high-throughput sequencing of DNA and RNA. This technological advancement has driven numerous omics applications, transforming fields such as oncology and agriculture. In oncology, NGS is extensively used to identify genetic mutations in tumors, aiding in the development of targeted therapies (Horak *et al.* 2016). Moreover, during the Corona Virus Disease 2019 (COVID-19) pandemic, NGS played a crucial role in identifying and tracking the severe acute respiratory syndrome coronavirus 2 (SARS-CoV-2) virus (Chiara *et al.* 2021). Beyond medical applications, NGS is utilized in agriculture to improve yields, quality, and resistance by studying plant genomes (Sun *et al.* 2022).

With the widespread adoption of NGS technology, the volume of data generated has increased exponentially, leading to challenges in data management and analysis. To support global scientific research, several organizations, including the International Nucleotide Sequence Database Collaboration (INSDC) (Arita *et al.* 2021) and the Genome Sequence Archive (GSA) (Chen *et al.* 2021), have been established to collect and disseminate nucleotide sequence data and associated metadata. These organizations maintain vast amounts of NGS data, facilitating access and collaboration within the scientific community. Additionally, these organizations use a standardized data model to harmonize data accession numbers across various sources, greatly enhancing the management, sharing, and retrieval of NGS data.

Despite these advancements, the growing volume of NGS data continues to present challenges for efficient retrieval and analysis. To address these challenges, we introduce iSeq, a tool designed to streamline the retrieval of metadata and NGS data from major databases, including GSA, SRA, ENA, and DDBJ. iSeq aims to enhance data accessibility by supporting various accession formats and providing advanced features such as parallel downloads and multi-threaded processing. This tool is intended to facilitate more efficient data management and analysis, thereby accelerating scientific discoveries and applications in diverse fields.

## 2 Materials and methods

**Figure 1.**
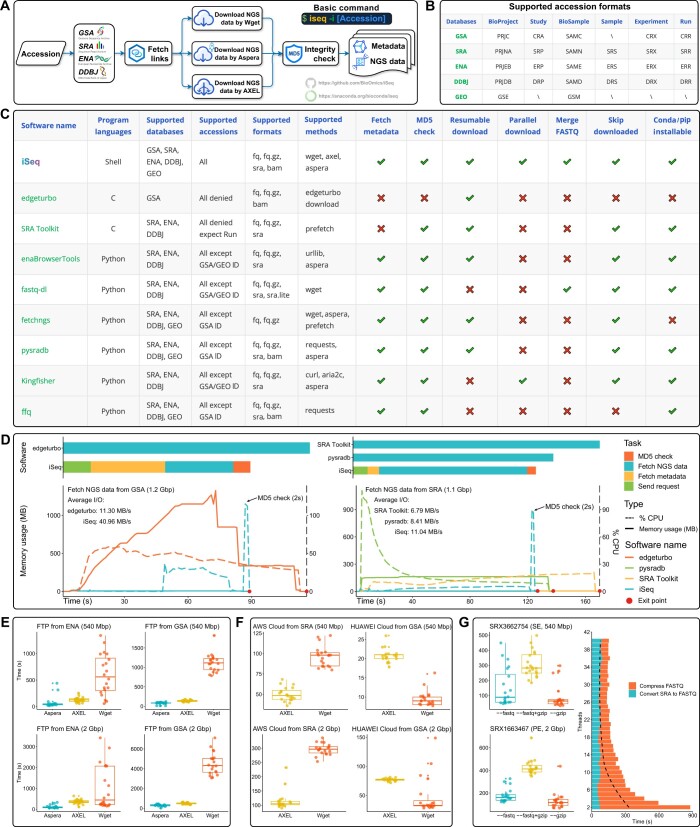
Comparison of iSeq with existing tools and performance benchmarks. (A) Overview of the iSeq design. (B) Supported accession formats in iSeq. (C) Comparative analysis of iSeq versus other tools. (D) Bar and line plots showing execution time, memory usage, CPU utilization, and average I/O for iSeq compared to alternative tools. (E) Download speed comparison using different methods within iSeq. (F) Cloud storage download speed comparison between AXEL and Wget methods in iSeq. (G) Efficiency of downloading gzip-formatted FASTQ files using varying thread counts.

iSeq is a Bash script designed to efficiently fetch public sequencing data from multiple bioinformatics databases, including GSA, SRA, ENA, and DDBJ. The workflow begins by providing an accession number (Fig. 1A), which can be in various formats such as Project, Study, Sample, Experiment, or Run. iSeq automatically detects the accession format and fetches metadata from the appropriate source, prioritizing ENA among the partner organizations of INSDC or GSA due to their extensive data availability.

Once the metadata is fetched, iSeq determines the best source for downloading the sequencing data. If the fetched accession is in the INSDC databases and the FTP channel is available in ENA, iSeq will proceed to download the data using either Aspera (https://www.ibm.com/products/aspera) for fast transfer, Wget (https://www.gnu.org/software/wget), or AXEL (https://github.com/axel-download-accelerator/axel) for parallel downloading. In cases where the FTP channel is not available in ENA, iSeq will fetch download links from AWS Cloud channel, again utilizing Wget or AXEL based on user preferences. If data are stored in GSA and the HUAWEI Cloud channel is accessible, iSeq prioritizes accessing the HUAWEI Cloud channel for its speed and reliability. Alternatively, iSeq will use the FTP channel with Aspera, Wget, or AXEL for downloading. After the initial download, iSeq will conduct an MD5 checksum to ensure the integrity of the downloaded files. If the checksum does not match the expected value in public databases, iSeq will attempt to redownload the files up to three times. Files that pass the MD5 check are logged as successfully downloaded, while those that fail after three attempts are recorded in a failure log.

For users requiring compressed data, iSeq can directly download FASTQ files in gzip format if available. Otherwise, iSeq will download SRA files and converts them to FASTQ format using fasterq-dump (https://github.com/ncbi/sra-tools), followed by compression with pigz (https://zlib.net/pigz), which is only considered when fetching from INSDC-related databases. For the GSA database, iSeq always directly fetches compressed or BAM format data. Additionally, iSeq can merge multiple FASTQ files from the same Experiment, Sample, or Study into a single file for single-end sequencing data, or maintain the order and consistency of read names in two files for paired-end sequencing data. If the Experiment, Sample, or Study consists of only one run, the Run accession will be renamed in the format of the Experiment, Sample, or Study accession. Finally, upon successful execution, iSeq will generate the corresponding metadata and NGS data.

## 3 Results

The exponential growth of publicly stored NGS data necessitates effective retrieval tools. Drawing inspiration from existing tools such as fastq-dl, fetchngs (Ewels *et al.* 2020), pysradb (Choudhary 2019), ffq (Gálvez-Merchán *et al.* 2023), and Kingfisher, we developed iSeq to provide enhanced functionality and comprehensive database support, including GSA, SRA, ENA, and DDBJ (Fig. 1A). iSeq now supports 25 different accession formats as inputs (Fig. 1B). iSeq’s unique features include support for GSA database and various NGS data formats, such as FASTQ, gzip-formatted FASTQ, SRA, and BAM (Fig. 1C). Notably, iSeq avoids SRA Lite format due to its potential impact on downstream analyses. Additionally, iSeq integrates Aspera for fast data retrieval and supports multi-threaded downloads using AXEL. iSeq performs integrity checks and includes breakpoint reconnection functionality to ensure accurate data retrieval.

To evaluate iSeq’s performance, we tested its ability to download 3000 gzip-formatted FASTQ files (∼7 Terabyte of base pairs, Tbp) from GSA and 3000 SRA files (∼5 Tbp) from INSDC-related databases. Results showed high success rates and integrity for these downloads (Supplementary Table S1). To comprehensively evaluate the performance of various tools, we conducted tests under consistent conditions: utilizing the same computing cluster (48 cores, 128GB RAM), at the same time, and with identical network speeds (download/upload = 1000/1000 Mbps). Our results indicate that iSeq outperforms in terms of faster execution time, lower memory consumption, and greater scalability, especially when handling datasets of varying sizes (Fig. 1D). iSeq’s efficiency in fetching data through various channels was also assessed. Aspera provided the fastest download speeds for INSDC-related databases, while AXEL achieved comparable speeds for GSA FTP channels (Fig. 1E). For cloud-stored data, iSeq uses AXEL and Wget. AXEL achieved faster speeds for AWS Cloud data from SRA, whereas Wget was slightly faster for HUAWEI Cloud data from GSA (Fig. 1F). iSeq’s ability to handle gzip-formatted FASTQ files efficiently was demonstrated, with direct fetching proving to be the most effective method (Fig. 1G). The tool’s capability to handle multiple threads improved compression speed but had limited impact on decompression speed when threads exceeded 15 (Fig. 1G).

In summary, iSeq enables faster and more stable downloads for FTP channel data through the “--aspera” parameter and supports parallel downloads for cloud-stored data with the “--parallel” parameter. It is important to note that actual download speeds may still vary depending on the user’s network environment and the real-time throughput of public databases.

## Supplementary Material

btae641_Supplementary_Data

## Data Availability

NGS data utilized in this study are publicly available from various repositories. Specifically, experiments SRX3662754 and SRX1663467 were retrieved from ENA and SRA, respectively. Additionally, experiments CRX917377 and CRX095512 were obtained from GSA. iSeq is accessible to all researchers via GitHub (https://github.com/BioOmics/iSeq) or Bioconda (https://anaconda.org/bioconda/iseq). The code and data used for performance benchmarks can also be accessed on GitHub (https://github.com/BioOmics/iSeq/tree/main/docs/benchmark).
